# SMAD proteins directly suppress *PAX2* transcription downstream of transforming growth factor-beta 1 (TGF-β1) signalling in renal cell carcinoma

**DOI:** 10.18632/oncotarget.25516

**Published:** 2018-06-01

**Authors:** Gagandeep Kaur, Caiyun Grace Li, Andrew Chantry, Cherie Stayner, Julia Horsfield, Michael R. Eccles

**Affiliations:** ^1^ Department of Pathology, Dunedin School of Medicine, University of Otago, Dunedin, New Zealand; ^2^ School of Biological Sciences, University of East Anglia, Norwich, United Kingdom

**Keywords:** TGF-β1 signalling, PAX2 expression, epithelial to mesenchymal transition, clear cell renal cell carcinoma, SMAD proteins

## Abstract

Canonical TGF-β1 signalling promotes tumor progression by facilitating invasion and metastasis, whereby release of TGF-β1, by (for example) infiltrating immune cells, induces epithelial to mesenchymal transition (EMT). PAX2, a member of the Paired box family of transcriptional regulators, is normally expressed during embryonic development, including in the kidney, where it promotes mesenchymal to epithelial transition (MET). PAX2 expression is silenced in many normal adult tissues. However, in contrast, PAX2 is expressed in several cancer types, including kidney, prostate, breast, and ovarian cancer. While multiple studies have implicated TGF-β superfamily members in modulating expression of *Pax* genes during embryonic development, few have investigated direct regulation of *Pax* gene expression by TGF-β1. Here we have investigated direct regulation of *PAX2* expression by TGF-β1 in clear cell renal cell carcinoma (CC-RCC) cell lines. Treatment of *PAX2*-expressing 786-O and A498 CC-RCC cell lines with TGF-β1 resulted in inhibition of endogenous *PAX2* mRNA and protein expression, as well as expression from transiently transfected *PAX2* promoter constructs; this inhibition was abolished in the presence of expression of the inhibitory SMAD, SMAD7. Using ChIP-PCR we showed TGF-β1 treatment induced SMAD3 protein phosphorylation in 786-O cells, and direct SMAD3 binding to the human *PAX2* promoter, which was inhibited by SMAD7 over-expression. Overall, these data suggest that canonical TGF-β signalling suppresses *PAX2* transcription in CC-RCC cells due to the direct binding of SMAD proteins to the *PAX2* promoter. These studies improve our understanding of tumor progression and epithelial to mesenchyme transition (EMT) in CC-RCC and in other *PAX2*-expressing cancer types.

## INTRODUCTION

Approximately 30 Members of the TGF-β superfamily, including TGF-βs, activins, bone morphogenic proteins (BMPs), anti-Mullerian hormone (MIS/AMH), and growth/differentiation factors (GDFs), such as myostatin, represent multifunctional cytokines secreted by various cell types. TGF-β superfamily ligands regulate a vast array of biological processes such as growth, differentiation, migration, extracellular matrix production, angiogenesis, cytokine secretion, apoptosis, setting up of the body plan and organogenesis during embryogenesis, tissue homeostasis and immune regulation in adults [1–7]. Disruption of TGF-β signalling has been implicated in the progression of diseases such as cancer, fibrosis and autoimmune disease [8].

The primary signalling pathway downstream of TGF-β superfamily receptor/ligand interactions (for instance, interaction of TGF-β1 with type I and type II TGF-β receptors [9], and of BMPs with type I and type II serine/threonine kinase receptors [10, 11]) involves activation of Smad transcription factors [12–16]. In response to receptor/ligand interactions, Smad2 or Smad3 proteins become phosphorylated and form homomeric and heteromeric complexes with co-Smads, after which the complexes translocate to the nucleus where they either directly bind DNA or co-operate with sequence specific transcription factors/DNA binding proteins to regulate transcription of genes either positively or negatively [17]. In opposition to Smad2, or Smad3 effector signalling, the vertebrate I-Smads, Smad6 and Smad7 act as potent antagonists of TGF-β signalling [18–23] through recruitment of either ubiquitin ligases (Smurf1/2), or protein phosphatase I, or by becoming part of the TGF-β receptor complex and thereby interfering in the formation of SMAD2/SMAD3/co-SMAD complexes.

In cancer, TGF-β1 plays a dual role, involving both tumor suppressor and oncogenic functions, depending on context [24]. For instance, in the early stages of tumorigenesis the TGF-β signalling pathway induces growth arrest and promotes apoptosis, and mutation or deletion of members of the TGF-β signalling pathway define a tumor suppressor role for TGF-β signalling [8]. In contrast, activation of the TGF-β signalling pathway occurs in the late stages of tumorigenesis, as tumor cells become more invasive and prone to metastases, which is accompanied by induction of epithelial to mesenchymal transition (EMT).

During kidney development, ectopic expression of Activin A, a member of the TGF-β superfamily, inhibits GDNF-induced ureteric bud outgrowth and cell proliferation [25], and reduces *Pax2* expression [26]. In contrast, BMP-7 induces *Pax2* expression during kidney development [2]. Furthermore, exogenous expression of TGF-β1 in dysplastic kidney epithelial-like cells, decreased expression of *Pax2*, which was associated with decreased proliferation and transition to a mesenchymal phenotype [27]. In addition, TGF-β1 treatment caused suppression of *Pax2* expression in proximal renal tubule cells [28]. Taken together, these data suggest there is an interaction between TGF-β1 signalling and *Pax2* in kidney development and disease.

*PAX2* is the second member of the *Paired box* (*Pax*) gene family, which has critical roles during early patterning events of the embryo [29, 30]. *PAX2* is expressed at a very early stage in the nephric lineage and is required for normal kidney development in both mice [31, 32] and humans [33]. In early kidney development, the induction and conversion of metanephric mesenchyme to nephrogenic epithelium has been shown to require *Pax2* activity during mesenchymal to epithelial transition (MET) [32], and this mechanism is also thought to operate during kidney regeneration and following kidney injury [34]. While *Pax2* is only transiently expressed in the nephrogenic mesenchyme of the kidney, unabated expression of *Pax2* leads to abnormal kidney hyperplasia and cystogenesis [35].

*PAX2* is expressed in multiple different cancer types, including in renal cell carcinoma (RCC), ovarian cancer, endometrial carcinoma, breast cancer, and prostate cancer, [30]. Of these various cancer types, some subtypes frequently express *PAX2* at high levels, whereas in the surrounding normal adult tissue the *PAX2* expression is repressed following cessation of development [30]. In serous ovarian cancer cells wild-type p53 was shown to activate *PAX2* expression [36]. However, the mechanisms by which *PAX2* expression is regulated in cancer remain relatively poorly understood, and in particular the mechanisms associated with repression of *PAX2* expression. For example, PAX2 protein expression has been noted at relatively early stages of tumor formation in serous ovarian carcinoma, but at later stages of progression the acquisition of metastasis is accompanied by loss of PAX2 expression [36]. Concomitantly, the loss of epithelial differentiation is associated with increased levels of TGF-β1 and TGF-β signalling in higher grades of ovarian cancer [37]. In serous ovarian carcinoma, down-regulation of *PAX2* expression during later stages of tumor development in secretory cell outgrowths (SCOUTs) has been identified, and members of the TGF-β downstream signalling pathway were expressed in the same cells [38, 39].

In RCC, loss of VHL and hypoxia has been shown to activate *PAX2* expression [40], but the potential role of *PAX2* expression in RCC at later stages of tumor development is less well understood. *PAX2* expression is also associated with several other kidney abnormalities, such as renal interstitial fibrosis [41] and polycystic kidney disease [42]. Following kidney injury, generation of renal fibrosis or scar tissue is dependent on the expression of TGF-β [43] and eventually, EMT-dependent suppression of PAX2 expression following its transient activation [41, 44]. Moreover, PAX2 expression in RCC has been shown to promote the expression of *ADAM10*, which is a negative regulator of EMT, and suggests further an association between PAX2 and suppression of EMT in RCC [45].

In this study we investigated the potential role of TGF-β1 signalling in promoting down-regulation of *PAX2* expression in RCC tissue. We have identified a direct role for SMAD proteins in binding to the *PAX2* gene promoter to suppress *PAX2* expression in human clear cell renal cell carcinoma (CC-RCC) cells, during canonical TGF-β signalling. This finding suggests there is a direct relationship between TGF-β1 signalling and *PAX2* expression during RCC tumor progression, with the implication that direct suppression of *PAX2* expression in some cancer cell types by TGF-β1 signalling is a corollary of the promotion of EMT, tumor invasion and metastasis.

## RESULTS

### Treatment of RCC cancer cell lines with TGF-β1 leads to suppression of *PAX2* mRNA and protein levels

**Figure 1:**
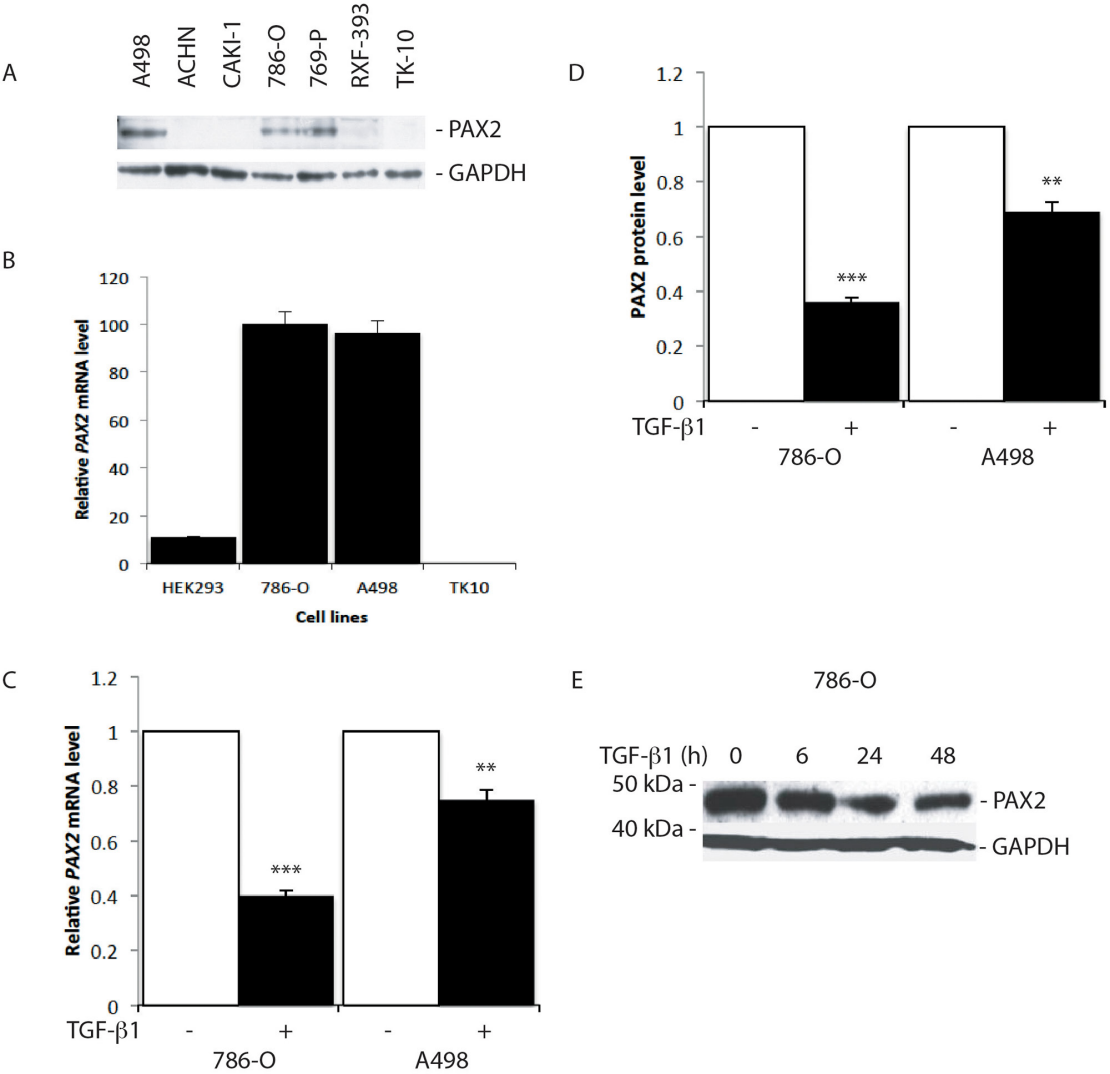
TGF-β1 treatment suppresses *PAX2* mRNA and protein expression in CC-RCC cell lines **(A)** Western blot analysis of PAX2 protein expression in RCC cell lines. GAPDH was used as a loading control. **(B)** QRT-PCR analysis of the relative level of endogenous *PAX2* mRNA in HEK293 and three CC-RCC human cell lines. The data represent three separate experiments. **(C)** Relative *PAX2* mRNA expression level, and **(D)** PAX2 protein following treatment of 786-O and A498 CC-RCC cells with 10 ng/ml TGF-β1 (labelled “+”, black columns) versus vehicle treated controls (labelled “-“, white columns). The results from three separate experiments after normalisation with GAPDH are shown. **(E)** Western blot of PAX2 protein expression relative to GAPDH following no treatment (0 h) of 786-O cells, or treatment for 6, 24 or 48 h with 10 ng/ml TGF-β1. Data are represented as mean ± S.E.M. The data were analysed by Student's *t* test using GraphPad Prism 5.01, ^**^ p < 0.01, ^***^ p < 0.001 versus controls.

Treatment of *PAX2* expressing RCC cell lines, 786-O and A498 (Figure 1A, 1B), with 10 ng/ml TGF-β1 for 24 h resulted in substantial suppression of *PAX2* mRNA and protein levels (Figure 1C-1E), and further analysis revealed significant inhibition of *PAX2* mRNA and protein expression in 786-O cells in response to increasing concentrations of TGF-β1, which was sustained from 6 h to more than 48 h after treatment (Figure 1E, Supplementary Figure 1). The results of these investigations are consistent with previous studies, suggesting that TGF-β signalling pathways inhibit *Pax* gene expression [46].

### TGF-β1 treatment suppresses *PAX2* promoter activity in RCC cells

**Figure 2:**
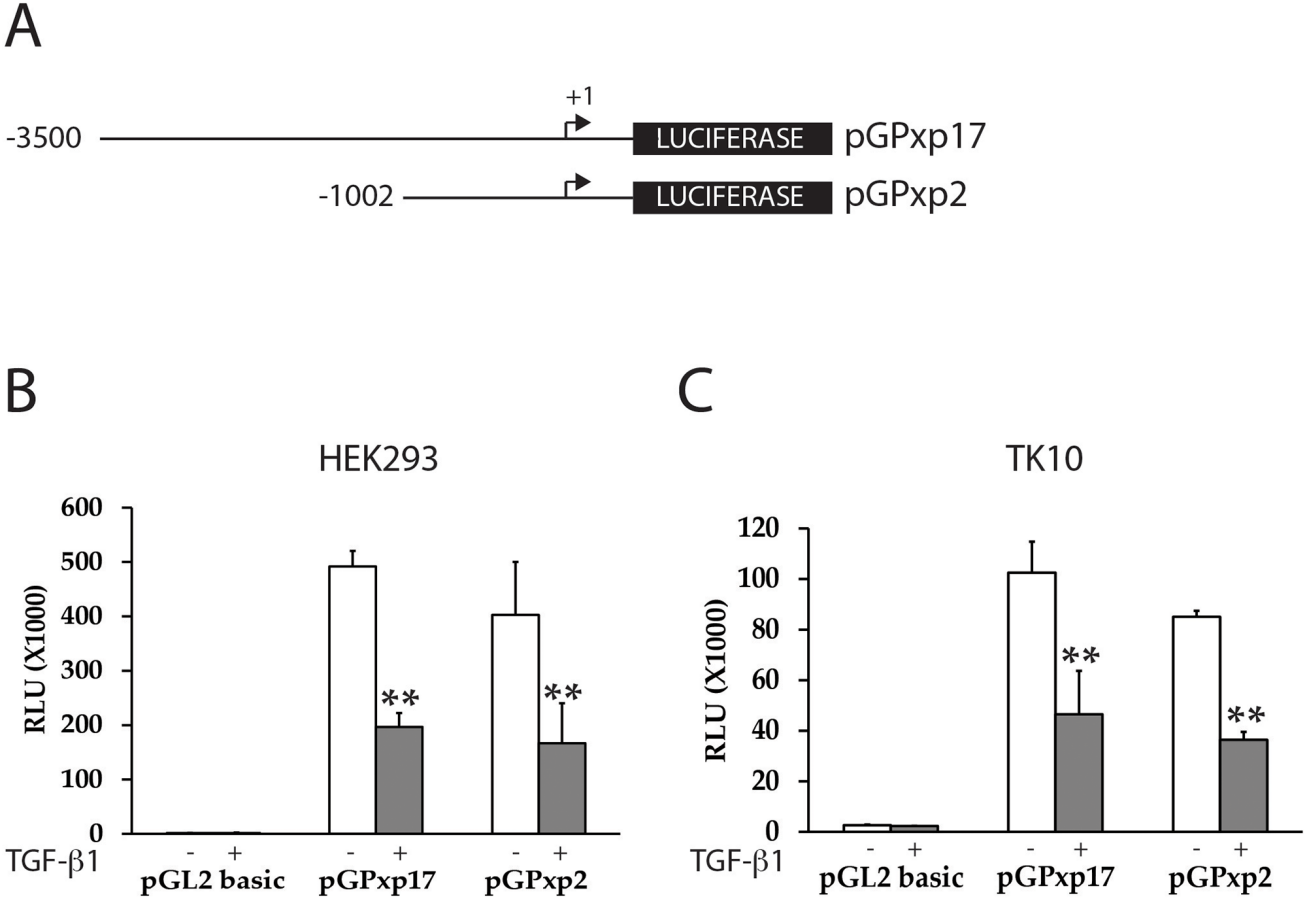
TGF-β1 treatment suppresses *PAX2* promoter activity in CC-RCC cell lines **(A)** The *PAX2* promoter reporter constructs (pGPxp17 and pGPxp2) are shown. **(B)** HEK293 or **(C)** TK10 cells were transiently transfected with *PAX2* promoter constructs (pGPxp17 or pGPxp2) and treated with 10 ng/ml TGF-β1 and the luciferase activity was assayed in TGF-β1 treated (grey columns) versus cells treated with vehicle control (white columns) The data represent the mean ± S.E.M of at least three experiments and were analyzed by Student's *t* test (^**^, p < 0.01). HEK293 (n=5), TK10 (n=4).

To determine whether TGF-β treatment mediates inhibition of PAX2 expression directly or indirectly via regulation of the *PAX2* promoter, we investigated whether TGF-β1 treatment could suppress transiently transfected human *PAX2* promoter constructs in kidney and RCC cell lines. Previous studies have shown that there is potential for positive transcriptional auto-activation on *PAX* gene promoters by PAX proteins [46, 48]. We therefore reasoned that it would be important to minimize any potential confounding effects of auto-regulation on TGF-β1- mediated PAX2 suppression. HEK293 (human embryonic kidney), or TK10 (CC-RCC) cell lines have previously been shown to express little or no endogenous *PAX2* mRNA or protein [47] (Figure 1). We found that *PAX2* promoter-luciferase reporter constructs (pGPxp17 and pGPxp2, Figure 2A) were transcriptionally active in the HEK293 and TK10 cell lines (Figure 2), suggesting that these cells would be suitable to carry out the reporter assays. The pGPxp17 and pGPxp2 promoter-reporter constructs were transiently transfected into the HEK293 and TK-10 cells, which were then treated for 24 h with 10 ng/ml TGF-β1. Luciferase reporter activity was suppressed by the TGF-β1 treatment (Figure 2B, 2C), suggesting that TGF-β1 treatment caused transcriptional suppression of the *PAX2* promoter construct activity in the transient transfection assays.

### Expression of SMAD-7 in RCC cells abolishes the inhibitory effect of TGF-β1 on PAX2 protein and mRNA expression

**Figure 3:**
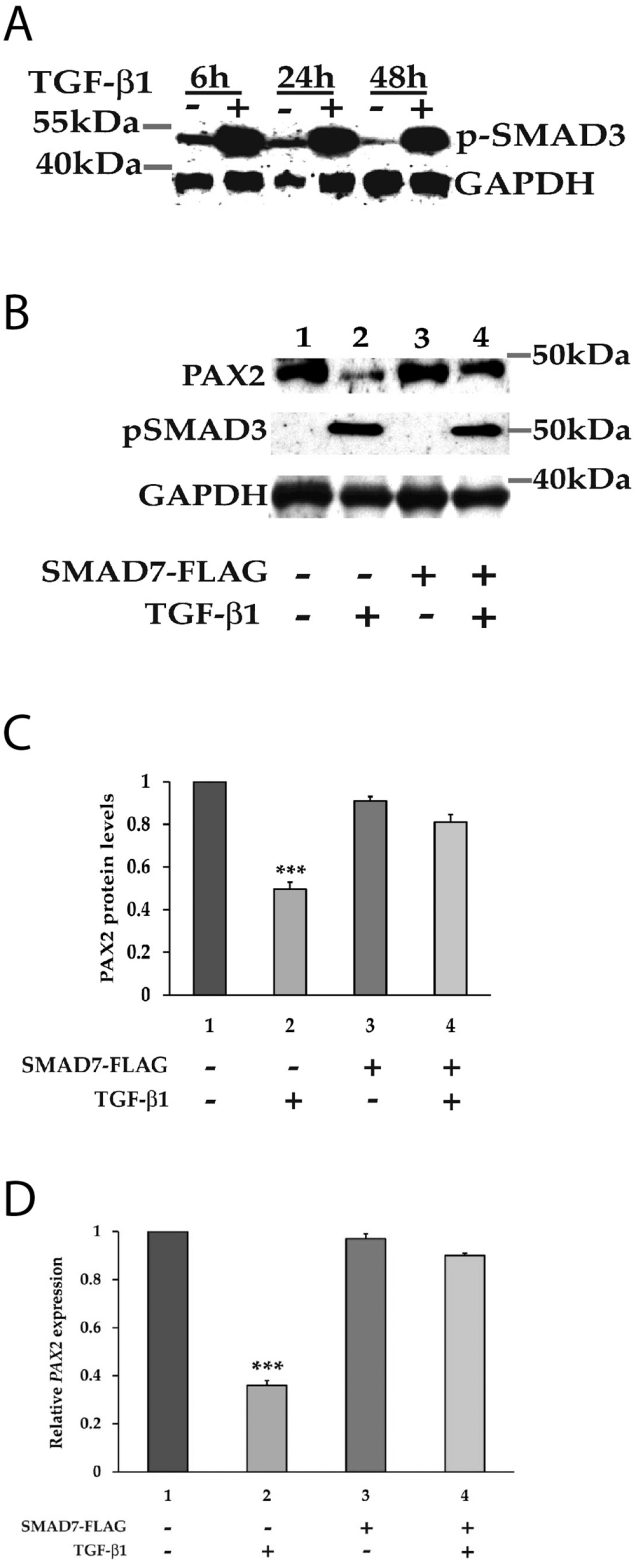
The canonical SMAD signalling pathway is involved in the regulation of *PAX2* by TGF-β1 in 786-O cells **(A)** Following treatment with TGF-β1, downstream SMAD3 becomes phosphorylated at carboxyl terminal serine residues. The phosphorylation of SMAD3 was determined in vehicle (-) and TGF-β1 (+) treated 786-O cells at 6 h, 24 h and 48 h post stimulation using phospho-Smad3 antibody, which detects endogenous levels of Smad3 when phosphorylated at Ser423/425. GAPDH served as a loading control. **(B)** Phosphorylation of SMAD3 upon treatment with TGF-β1 was associated with inhibition of PAX2 expression, and PAX2 suppression was abrogated upon expression of the iSMAD, SMAD7. **(C)** Quantitation of the PAX2 protein levels and **(D)** *PAX2* mRNA levels in 786-O cell as treated in (B) in two separate experiments after normalization with GAPDH or with housekeeping genes relative to vehicle treated cells (with the PAX2 expression level set to 1). The data are represented as mean ± S.E.M. and were analyzed by one-way ANOVA followed by Tukey's multiple comparison post-test (^***^, p < 0.001 versus control cells). The first column represents cells with no transfection and the dark grey (column 3) represents cells transiently transfected with the SMAD7-FLAG expression construct, while the lighter grey (columns 2 and 4) represent respective cells treated with TGF-β1.

We next investigated whether signalling via SMAD proteins, which are known to mediate canonical TGF-β1 signalling, could be involved in the transcriptional suppression of *PAX2* by TGF-β1. Exposure of 786-O cells to TGF-β1 for 6 h, 24 h or 48 h led to the strong phosphorylation of SMAD3 protein (Figure 3A), consistent with the expected effects of TGF-β1 treatment. Evidence for the involvement of SMAD proteins in the regulation of PAX2 expression was obtained by expressing an inhibitory SMAD (SMAD7), which we predicted would abolish the suppressive effect of TGF-β signalling on PAX2 expression levels if indeed SMAD signalling was involved in suppressing *PAX2* transcription. To investigate this, 786-O cells were transiently transfected with an inhibitory SMAD7 expression construct, or an empty expression vector, then treated with either TGF-β1 or vehicle 24 h post-transfection. After transfection the cells were analyzed for relative levels of PAX2 protein and mRNA expression a further 48 h later. The treatment of 786-O cells with TGF-β1 suppressed PAX2 protein levels by 50 +/- 0.03% (p<0.001) and *PAX2* mRNA by 60 +/- 0.03% (p<0.001) in the absence of SMAD7 (Figure 3B, lane 2), but in the presence of SMAD7, TGF-β1 treatment did not significantly suppress either levels of PAX2 protein (Figure 3B, lane 4 and Figure 3C), or *PAX2* mRNA (Figure 3D). SMAD3 phosphorylation was observed following TGF-β1 treatment of 786-O cells, irrespective of the presence of SMAD7 expression (Figure 3B, lanes 2 and 4). These data suggest that SMAD proteins are directly associated with suppression of PAX2 expression, as the suppression of *PAX2* by TGF-β1 treatment, which was associated with phosphorylation of SMAD3, was overcome by overexpression of the inhibitory SMAD (SMAD7) in 786-O cells.

### Is there a physical interaction between PAX2 and SMAD2 or SMAD3?

**Figure 4:**
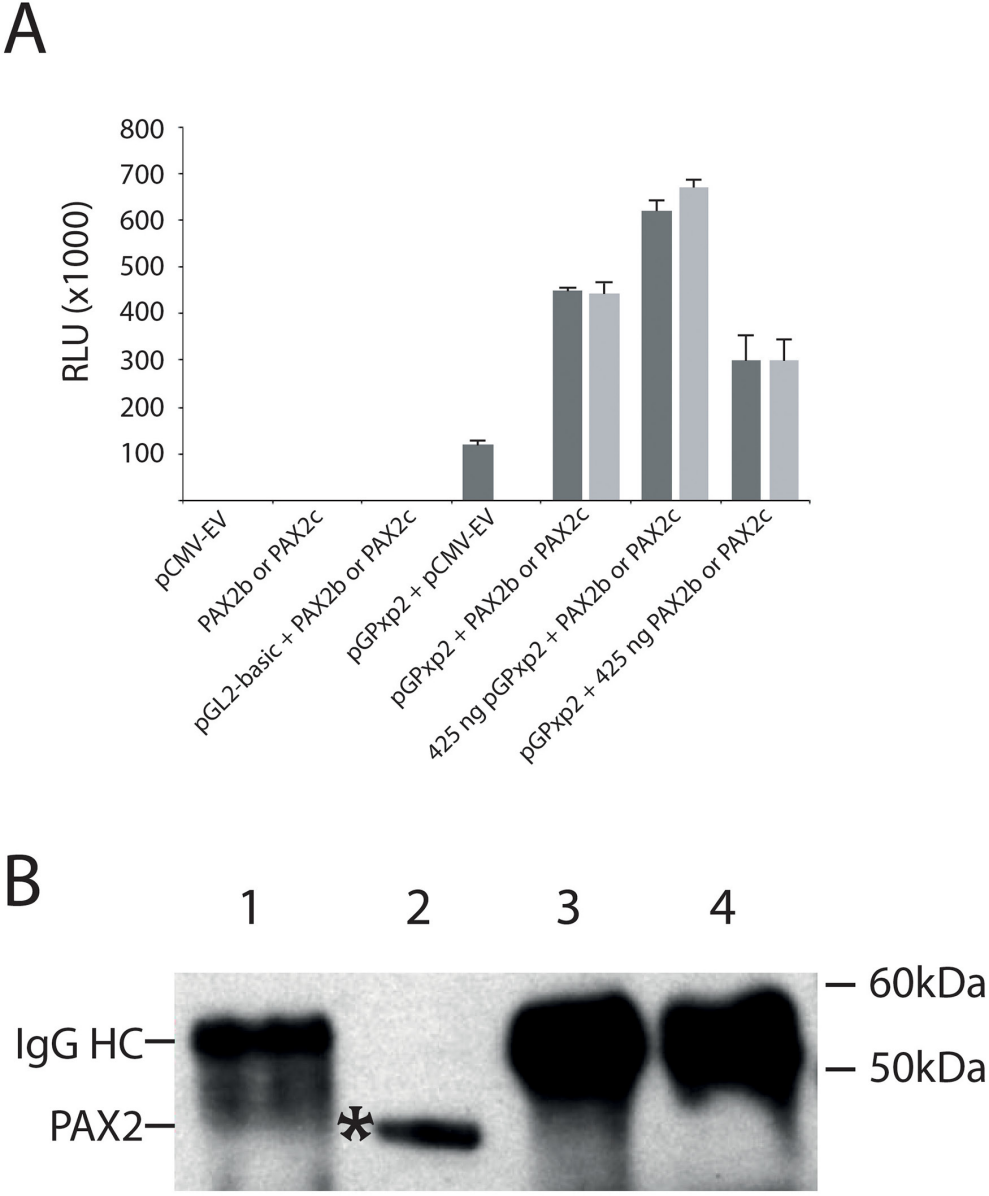
The PAX2 protein positively auto-regulates its own gene promoter, but does not physically interact with SMAD2/SMAD3 proteins **(A)** Demonstration of auto-regulation of the *PAX2* promoter in HEK293 cells. HEK293 cells were co-transfected with 150 ng of the PAX2 promoter plasmid (pGPxp2) together with pCMV-empty vector (“pGPxp2 + pCMV-EV”), which resulted in moderate luciferase activity (118,300 RLU). However, when HEK293 cells were co-transfected with 150 ng of *PAX2* promoter reporter construct, pGPxp2, together with 150 ng of *PAX2* expression construct, either pCMV-PAX2b (PAX2b, dark grey bar; 449,375 RLU), or pCMV-PAX2c (PAX2c, light grey bar; 444,159 RLU), there was 3.75-3.8-fold increased luciferase activity induced compared to pGPxp2 + pCMV-EV co-transfection. The graph summarizes three separate experiments for each co-transfection. The amounts of all *PAX2* promoter-reporter or *PAX2* expression construct plasmid that were used in the graph were 150 ng, unless otherwise specified. Error bars show the standard error of the mean. **(B)** Investigation of protein-protein interactions between PAX2 and SMAD2 or SMAD3. Lane 1: Eluate from beads used for pre-clearing the lysate. Lane 2: Input. Lane 3: Eluate from beads, which captured the protein complexes incubated with anti-IgG antibody. Lane 4: Eluate from beads, which captured the protein complexes incubated with anti-SMAD2/3 antibody. The figure shows that in lane 4, PAX2 was not immunoprecipitated with anti-SMAD2/3 antibody, suggesting there was no functional interaction between PAX2 and SMAD2 or SMAD3. The experiment was repeated twice. IgG HC: IgG heavy chain.

SMAD proteins have been shown to physically interact with PAX6 protein, preventing PAX6 from auto-activating its own gene promoter [46, 48]. We therefore next determined whether SMAD proteins interact with the PAX2 protein, but we first sought to determine whether PAX2 auto-activates its own promoter. HEK293 cells were transiently co-transfected with 150 ng of PAX2 promoter-luciferase reporter construct and PAX2 expression constructs. We observed increased PAX2 promoter-luciferase activity (~444,000 to 449,000 RLU for PAX2b and PAX2c, respectively) in these co-transfections compared to the co-transfection of the equivalent amount of *PAX2* promoter-luciferase reporter and empty vector expression constructs (~118,000 RLU, Figure 4A, 4B). When co-transfected promoter-luciferase reporter construct, pGPxp2, was increased to 425 ng, while maintaining PAX2b or PAX2c at 150 ng, this resulted in a further increase in luciferase activity (~620,000 to 670,000 RLU for PAX2b and PAX2c, respectively). In addition, when pGPxp2 was maintained at 150 ng, while increasing the PAX2b or PAX2c to 425 ng, this also resulted in increased luciferase activity (~300,000 RLU for either PAX2b and PAX2c). These results suggest that PAX2 auto-activates its own promoter. It is therefore possible that, like Pax6, SMAD proteins could interact directly with the PAX2 protein and thereby prevent auto-activation of the *PAX2* promoter, as had been demonstrated for TGF-β1-mediated regulation of Pax6 expression [46, 48]. To investigate this, we carried out co-immunoprecipitation using an anti-SMAD2/3 antibody to determine whether a direct protein-protein interactions occurs between SMAD2 or SMAD3 proteins and the PAX2 protein. No evidence was found for an interaction between SMAD2/3 and PAX2 proteins (Figure 4B). In contrast, there was evidence of a potential interaction between SMAD2/3 and PAX6 protein (Supplementary Figure 3), as reported previously [48].

### SMAD3 directly binds to the PAX2 promoter in RCC cells in response to TGF-β1 treatment

**Figure 5:**
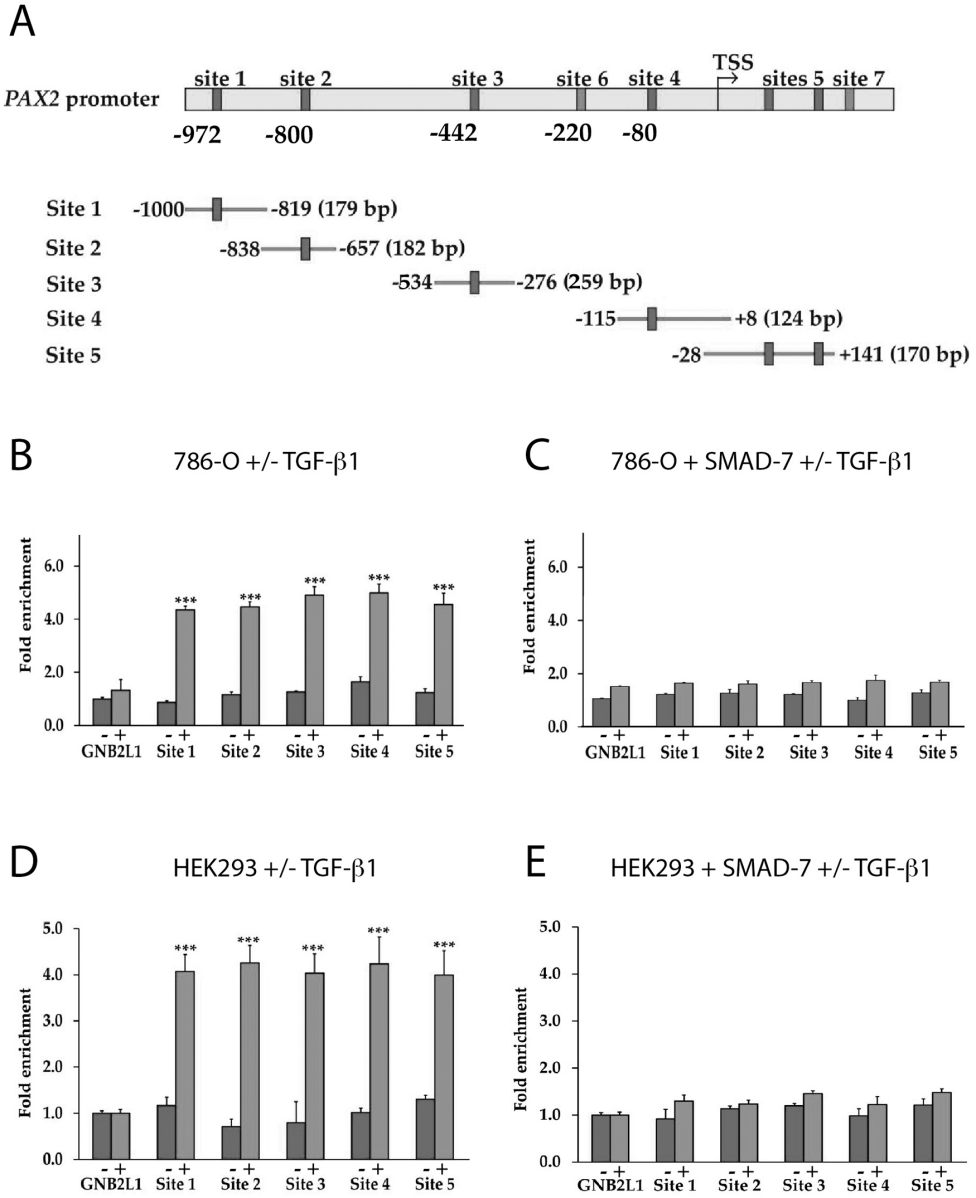
The binding of SMAD3 to the *PAX2* promoter in response to TGF-β1 treatment is inhibited in the presence of SMAD7 overexpression **(A)** Representation of the human *PAX2* promoter (not drawn to scale) showing SMAD1 or SMAD3 binding sites predicted using ConTra v2, and PCR fragments used for PCR-ChIP assays. Putative SMAD3 binding sites were identified at sites 4 and 6, while the remaining sites predicted by the ConTra binding site prediction tool were putative SMAD1 binding sites. The “TSS” shows the transcription start site. In **(B)** to **(E)** ChIP assays were carried out in 786-O and HEK293 cells following TGF-β1 treatment to show binding of SMAD3 to PCR-amplified fragments the *PAX2* promoter. Sites 6 and 7 in the *PAX2* promoter were unable to be amplified and were not analysed, but all other sites (sites 1-5) were investigated. In parallel experiments ChIP assays were carried out in cells transfected with a SMAD7-FLAG expression construct (see Supplementary Figure 2 for confirmation of SMAD7 overexpression in 786-O and HEK293 cells). A region of the *GNB2L1* promoter without any putative SMAD3 binding site was used as a negative control. Dark grey columns represent fold enrichment (over no antibody controls) in cells stimulated with vehicle and lighter grey columns represent fold enrichment in cells stimulated with TGF-β1. The data represent the mean ± S.E.M of three independent experiments and were analyzed by one-way ANOVA followed by Tukey's multiple comparison post-test (^***^, p < 0.001 versus negative control and vehicle treated cells).

We next investigated whether SMAD3 could bind directly to the PAX2 promoter. Using the binding site prediction tool, ConTra v.2, at least seven SMAD (SMAD1 or SMAD3) binding sites were predicted in the ~1Kb of the *PAX2* promoter upstream of the transcription start site (TSS), or in the 5’ untranslated region (5’UTR) (Figure 5A). Promoter-ChIP-PCR binding assays were used to investigate the binding of SMAD2 or SMAD3 proteins directly to the *PAX2* promoter following TGF-β1 treatment in both 786-O and HEK293 cells, and an approximately 4 to 5-fold enrichment of SMAD2/3 binding to *PAX2* promoter fragments was observed (p<0.001) (Figure 5B, 5D). Although the individual binding sites could not be resolved by the binding of SMAD proteins in the *PAX2* promoter in ChIP analysis, since individual sonicated fragments were between 200-800 bp in length and potentially encompassed multiple SMAD binding sites, the results nevertheless suggested that significantly increased binding of SMAD proteins to the *PAX2* promoter occurred in cells treated with TGF-β1. As a negative control the *GNB2L1* gene promoter was investigated, which contains no SMAD binding site in its promoter, and no enrichment of SMAD2 or SMAD3 binding was observed following TGF-β1 treatment in either 786-O or HEK293 cells (Figure 5B, 5D). We then investigated the effects of the inhibitory SMAD, SMAD7 on binding of SMAD2/3 to the *PAX2* promoter. The 786-O or HEK293 cells were transiently transfected with a SMAD7 expression construct, and the promoter-ChIP-PCR binding assays were again carried out. Following expression of SMAD7, the SMAD2/3 binding was inhibited (Figure 5C, 5E). These ChIP-PCR results suggest that SMAD3 protein is able to bind directly to DNA fragments within a 1Kb region of the *PAX2* promoter in ChIP-PCR binding assays, and that this binding is inhibited upon overexpression of SMAD7.

## DISCUSSION

Here we show that TGF-β1 treatment of renal cell carcinoma cells is associated with transcriptional supression of *PAX2*, through the direct binding of SMAD proteins to the *PAX2* promoter. Although our ChIP data demonstrate binding of SMAD3 to the *PAX2* promoter, these results do not distinguish which sites are functional, and further work will be required to determine this. Nevertheless, these findings represent the first evidence for direct promoter-mediated inhibition of *PAX2* expression by TGF-β1 signalling in tumor cells. With respect to several other *Pax* family members, it has previously been shown that TGF-β signalling regulates *Pax6* or *Pax8* gene expression through protein-protein interactions between SMAD2/3 and the Pax protein, resulting in inhibition of auto-activation of the respective *Pax* gene promoter [48, 49]. In contrast, our findings suggest that, in the case of *PAX2* expression in CC-RCC, SMAD2/3 proteins directly bind to the gene promoter, resulting in *PAX2* transcriptional supression.

Many previous studies have shown that SMAD3 binding to gene promoters is often associated with target gene activation. While the mechanism by which the binding of SMADs to the *PAX2* promoter leads to transcriptional suppression in CC-RCC cells has not been elucidated, SMAD proteins have been shown to interact with, and are able to form transcriptionally inactive complexes with co-repressors in kidney cells [50]. It is therefore possible that in CC-RCC cells the binding of SMADs to the *PAX2* promoter interferes with factors transcriptionally activating the promoter, which then leads to suppression of promoter activation, similar to the suppression of C/EBPβ- and STAT3-mediated transcriptional activation of the haptoglobin promoter by SMAD proteins, which has previously been reported [51]. However, it is presently unknown which factors are involved in generating the *PAX2* promoter activity observed *in vitro* in RCC cells.

The findings reported here are of potential importance in understanding the role of *PAX2* expression in cancer progression and metastasis. Dysgulated expression of *PAX2* has been linked to several cancer types, including RCC, ovarian cancer, endometrial cancer, prostate cancer, breast cancer, melanoma, medulloblastoma and Wilms tumor [30]. While *PAX2* expression has been shown to promote tumor cell proliferation and survival in multiple cancer types, paradoxically in serous ovarian cancer it has been shown that at advanced tumor stages, with onset of invasion and metastasis, *PAX2* mRNA and protein expression decline, and eventually *PAX2* expression is lost in the invading cells [36]. This apparent selection against *PAX2* expression within subpopulations of tumor cells seems counter-intuitive to the notion that PAX2 plays an oncogenic role in tumorigenesis. However, it is possible that switching away from PAX2 expression might reflect characteristic physiological changes within the cells, associated with acquisition of invasive properties. In serous ovarian cancer, the down-regulation of PAX2 expression occurs in the secretory outgrowths (termed SCOUTs), in which aggressive changes have been documented to occur [38, 39, 52]. These outgrowths are associated with poorer patient prognosis and progression to metastasis. Interestingly, Pax2 is expressed normally in mouse adult oviduct tissue [47], and it has been shown that *Pax2* expression maintains the differentiation of adult mouse oviduct epithelium and inhibits the transition to a stem cell-like state [53]. Similarly in breast cancer, expression of PAX2 has been associated with a low invasive phenotype in luminal breast cancer cells [54], while in endometrial cancer joint loss of *PAX2* and *PTEN* expression was associated with progression to carcinoma [55].

PAX2 protein expression has been associated with 81-95% of CC-RCCs [56, 57], and we have also shown that *in vivo* tumorigenicity of RCC cells is reduced following siRNA-mediated knockdown of PAX2 expression [58]. Moreover, PAX2 protein is strongly expressed in primary CC-RCC in a high percentage (~66%) of tumor cells, and in CC-RCC metastases expression of PAX2 appears to be more intense and in a higher proportion (~80%) of tumor cells [57]. However, PAX2 expression has been shown to positively regulate expression of *ADAM10* in CC-RCC [45], and the suppression of *ADAM10* expression is associated with EMT, as well as up-regulation of Slug, and down-regulation of E-Cadherin expression [45]. In independent experiments we observed that, following siRNA-mediated silencing of PAX2 expression in A498 RCC cells, down-regulation of *ADAM10* mRNA levels occurred (unpublished data), which is in agreement with previously published work [45]. Down-regulation of *PAX2* expression as a result of enhanced TGF-β1 signalling in CC-RCC would thus be consistent with a role for *PAX2* in facilitating EMT-related changes at least in part via reduced *ADAM10* expression. Importantly, an increased level of TGF-β1 signalling has clearly been associated with increased invasion and metastatic changes in CC-RCC [59–63]. However, in contrast to ovarian cancer where PAX2 expression is reduced in SCOUTs, the high levels of PAX2 expression observed in metastatic sites of CC-RCC [57] seem to contradict the notion that decreased *PAX2* expression is associated with EMT in CC-RCC. Furthermore, it has been observed that *PAX2* expression levels are increased by hypoxia and VHL mutations in CC-RCC [40], and so further investigations will be required to determine how the TGF-β1/SMAD-mediated signalling pathway promotes invasion through modulation of *PAX2* expression in CC-RCC.

We propose that pathways involving TGF-β1 signalling, and subsequent down-regulation of *PAX2* expression facilitate the switching of tumor cells from PAX2-mediated epithelial cell differentiation to more stem cell-like mesenchymal programs. These are associated with EMT, and greater dependence on other signalling pathways, in which tumor cells have acquired increased motility, invasiveness and treatment resistance [36]. Suppression of *PAX2* expression with concomintant EMT-related changes might be applicable to multiple cancer types in which *PAX2* is expressed, including RCC, ovarian, breast, endometrial and prostate cancer. It is conceivable, however, that the therapeutic inhibition of *PAX2* could induce tumor progression and metastasis in some cancer types, particularly if continued expression of *PAX2* has a role in promoting epithelial differentiation and suppression of tumor metastasis in advanced stages of cancer. Although not investigated here, mechanisms involving TGF-β signalling, and suppressed expression of *PAX2* might also be the case in renal fibrosis, where a role for *PAX2* expression in kidney injury has also been identified [41]. In contrast, TGF-β–mediated suppression of PAX2 expression might not be universal to all cancer types. For example, increasing levels of PAX2 expression have been linked to metastatic progression in esophageal cancer [64].

In conclusion, our studies have demonstrated that TGF-β1 signalling in CC-RCC cells results in the direct inhibition of *PAX2* expression through SMAD-mediated transcriptional suppression of the *PAX2* gene promoter. These data therefore provide clearer understanding of the role of TGF-β1 signalling and *PAX2* expression in cancer.

## MATERIALS AND METHODS

### Cell cultures and TGF-β1 treatment

Cell lines used in this study were HEK293 (human embryonic kidney immortalized with adenovirus 5 DNA), A498, ACHN, CAKI-1, 786-O, 769-P, RXF-393, and TK10 (renal cell carcinoma cell lines) and were obtained from the Developmental Therapeutics Program, NIH (as part of the NCI-60 cell line panel), and cultured either in Dulbecco's Modified Eagle's Media (DMEM) (Invitrogen, USA) or in Roswell Park Memorial Institute (RPMI) 1640 medium (Invitrogen, USA) supplemented with 10% fetal bovine serum (FBS) at 37°C in a humidified environment with 5% CO_2_. Cells were seeded at 50-60% confluence, and in some experiments were incubated with recombinant human TGF-β1 (R & D systems, Minneapolis, MN) at a concentration of 10 ng/ml (whenever the concentration was not specified). Control cells were treated with vehicle (4 mM HCl containing 1 mg/ml bovine serum albumin). Cells were harvested at 24 h post TGF-β1 treatment for RNA extraction and at 48 h post TGF-β1 treatment for Western blot analysis.

### Plasmids and promoter reporter constructs

Luciferase reporter plasmids (pGPxp17 and pGPxp2) have been reported elsewhere [65], and contained the human *PAX2* gene promoter fused to the luciferase reporter gene of pGL2 basic vector (Promega, USA). The pGPxp17 and pGPxp2 plasmids contain 4.2 kb and 1.7 kb of the human *PAX2* promoter and 5’ – flanking sequences, respectively. Expression plasmids encoding PAX2 and inhibitory SMAD, SMAD7 (pSMAD7-FLAG) were described previously [66, 67]. A plasmid encoding β-galactosidase (βgal) under the CMV promoter (pCMV-βgal) was used as an internal control of transfection efficiency in all reporter assays. Plasmids used for transient transfections were prepared using Qiagen plasmid Midi/Maxi kits, according to the manufacturer's instructions (Qiagen, Chatsworth, CA).

### Luciferase reporter assays

Luciferase reporter assays were carried out following transient transfection of cells with promoter-luciferase reporter constructs as previously described [65]. Briefly, cells were plated in 24-well plates to give 30-50% confluence after 24 h, and transfections were carried out using FuGENE^®^ 6 Transfection Reagent (Roche Applied Science, Germany) according to the manufacturer's instructions. An optimal FuGENE (μl): DNA (μg) ratio of 6:1 was used for all cell types except TK10 cells for which the FuGENE: DNA ratio was 3:1. Control cells were transfected with an equal amount of pGL2 basic to measure background luciferase activity. Fifty nanograms of pCMV-βgal was co-transfected as an internal control for transfection efficiency and used for normalizing the data. Transfected cells were treated with 10 ng/ml TGF-β1 24 h after transfection, and cells were harvested at 48 h post TGF-β1 treatment. Luminescence was measured using the Synergy 2 Multi-Mode Microplate Reader (BioTek, USA) and Gen5 interface software (version 1.01.9).

### β-galactosidase activity assays

β-galactosidase assays were carried out as described previously [67]. Briefly, 10 μl lysate was incubated with 100 μl βgal substrate solution (200 mM sodium phosphate buffer pH 7.3, 2 mM MgCl_2_, 100 mM β-mercapthoethanol, 1.33 mg/ml ONPG (Sigma-Aldrich). The samples were incubated at 37°C until the appearance of a bright yellow colour. The absorbance of the reaction was measured at 410 nm, using the Synergy 2 plate reader.

### Total RNA purification and quantitative real-time PCR (qRT-PCR)

Total RNA for gene expression analysis was isolated as described previously [47]. Briefly, cells were lysed with 200 μl TRIzol reagent (Invitrogen, USA) per well of a 24-well plate. Total RNA was extracted using 1-bromo-3-chloropropane (BCP) (Molecular Research Centre, USA) and 70% ethanol (in DEPC-H_2_O) (ethanol, Sharlau, Spain; DEPC, Sigma-Aldrich, USA) then purified using the PureLink RNA Mini Kit (Invitrogen, USA) according to the manufacturer's instructions. 200 ng of total RNA from each sample was used for generating cDNA in a 20-μl reaction. First-strand cDNA was synthesized using a SuperScript III Reverse Transcriptase Kit (Invitrogen, USA), primed with dNTPs and 250 ng random hexamers (Invitrogen, USA), according to the enzyme manufacturer's instructions, and was performed at 25°C for 5min, 50°C for 1 hr, and 70°C for 15 min. Three microliters of first strand cDNA (diluted 1:10) and 0.2 μM gene-specific primers (Table 1) were used with the Platinum® SYBR® Green qPCR SuperMix UDG (Invitrogen, USA), to amplify and detect target genes. The qRT-PCR reactions were performed in an ABI-7300 Real time PCR system (Applied Biosystems, USA) in duplicates, using the following program: 50°C – 2 min, 95°C – 10 min, 45 cycles of 95°C – 15 sec and 60°C – 1 min, with an additional dissociation stage: 95°C – 15 sec, 60°C – 1 min, and 95°C – 15 sec. Relative gene expression data were normalised to three housekeeping genes, and represented relative to vehicle treated control cells. Gene-specific primers (Invitrogen) for qRT-PCR analysis were designed using Primer3 (version 0.4.0, http://frodo.wi.mit.edu/primer3/). In each QRT-PCR experiment, the three most stable housekeeping genes (also listed in Table 1) were identified.

**Table 1:** Oligonucleotide primers used for qRT-PCR

Target gene	Forward primer 5’- 3’	Reverse primers 5’ – 3’
***PAX* Genes**		
*PAX2*	CCTGGCCACACCATTGTTC	TCACGTTTCCTCTTCTCACCAT
***SMAD* Genes**		
*SMAD2*	TATGGACACAGGCTCTCCAG	CACCAAAATGCAGGTTCTGA
*SMAD3*	GCAGGTTCTCCAAACCTATCC	AGGAGATGGAGCACCAGAAG
**Housekeeping Genes**		
*GAPDH*	CTCAAGATCATCAGCAATGCC	GGTCATGAGTCCTTCCACGATAC
*GNB2L1*	CACAACGGGCACCACCAC	CACACACCCAGGGTATTCCAT
*HPRT1*	ATTATGGACAGGACTGAACGTCTTG	TGAGCACACAGAGGGCTACAAT
*RPL32*	AACGTCAAGGAGCTGGAAGTG	GGCTTTGCGGTTCTTGGA
*RPL13a*	GGAAAGAGAAAGCCAAGATCCA	GCTCAGACCAGGAGTCCGTG
*YWHAZ*	ACTTGACATTGTGGACATCGGATAC	GTTGGAAGGCCGGTTAATTTTC
*PPIB*	ATGATCCAGGGCGGAGACTT	CAGGCCCGTAGTGCTTCAG

### Total protein sample preparation, SDS-PAGE and western blotting

Protein isolation and Western blots were performed as previously described [68]. Briefly, cells were lysed in RIPA buffer (50 mM Tris-HCl pH 7.5, 150 mM NaCl, 1% NP-40, 0.5% sodium deoxycholate, 0.1% SDS supplemented with protease inhibitors (Complete, Roche; 1 mM PMSF, Sigma-Aldrich; 1mM sodium orthovandate, Sigma-Aldrich). Total protein in the extracts was quantitated using a colorimetric BCA Protein Assay Kit as per the manufacturer's instructions. 40 μg of total cellular protein for each sample was boiled in Laemmli sample buffer and separated by SDS-PAGE under reducing conditions and transferred to nitrocellulose membrane (Hybond-C Extra). Immunoreactive bands were visualized by chemiluminescence using SuperSignal West Pico Chemiluminescent Substrate (containing equal parts of the Stable Peroxide Solution and the Luminol/Enhancer Solution) (Thermo Scientific, USA). Antibodies used for Western blotting were rabbit anti-PAX2 (1:500; Zymed), mouse anti-SMAD2/3 (1:1000; Santa Cruz Biotechnology), goat anti-GAPDH (1:2000, Santa Cruz Biotechnology), and anti-rabbit and anti-mouse horseradish peroxidase IgG (Sigma-Aldrich). The protein ladder used was MagicMark XP Standard (Invitrogen, USA).

### Chromatin immunoprecipitation (ChIP)-PCR

ChIP-PCR assays were carried out as described in Sehgal *et al* (2009) [69] with several modifications. Briefly, approximately 1.0 × 10^8^ cells, with or without TGF-β1 treatment, were crosslinked per ChIP analysis. Following washing, scraping and centrifugation of the cells in ice cold PBS, the cells were then lysed in 50 mM Tris-HCl pH 8.0, 10 mM EDTA, 1% SDS supplemented with protease inhibitors (Complete, Roche; 1 mM PMSF, Sigma-Aldrich; 1mM sodium orthovandate, Sigma-Aldrich). The lysates were then sonicated to obtain DNA fragments in the range of 200-800 bp using a Sonics Vibra-Cell sonicator (Sonics and Materials Inc., USA, Model: VCX 130) set to 25% amplitude. HEK293 cells were sonicated for 5 sets of 10-second pulses and 786-O cells for 5 sets of 20-second pulses. Following centrifugation (12,000 x g, 10 min at 4°C) supernatants were diluted 2.5 times with ChIP dilution buffer (0.5% Triton X-100, 2 mM EDTA, 20 mM Tris-HCl pH 8.0, 100 mM NaCl), and 5% of the diluted supernatant was saved as an Input control. The rest of the supernatant was precleared using Dynabeads^®^ Protein G (Invitrogen) for 1 h at 4°C. For immunoprecipitation 4 μg of anti-SMAD2/3 (Santa Cruz Biotechnology) was added to precleared lysates and rotated at 4°C overnight. Lysates with no antibody served as a negative control. The remainder of the protocol was carried out essentially as described in Sehgal *et al* (2009). The qRT-PCR reactions were performed as described previously (ChIP PCR primers listed in Table 2) except an additional dissociation was performed at 99°C instead of 95°C. The data were analysed according to the SuperArray ChIP-qPCR Data Analysis Template (www.sabiosciences.com/manuals/chipqpcranalysis.xls). Individual binding sites were unable to be resolved by the binding of SMAD proteins in the *PAX2* promoter in ChIP analysis, because individual sonicated fragments were between 200-800 bp in length and potentially contained multiple SMAD binding sites.

**Table 2:** Oligonucleotide primers used for ChIP-PCR analysis

Target Gene	Accession no./Reference	Promoter region	Forward sequence 5’–3’	Reverse sequence 5’–3’
*PAX2*	4842, NM_000278	-997/-819, site 1	GGATCCACCGAGCTAGCAG	GGGACTTGGTTTCTGAACCC
*PAX2*	4842, NM_000278	-838/-657, site 2	GGGTTCAGAAACCAAGTCCC	GTCTTGTCCCCTCCCGTTCC
*PAX2*	4842, NM_000278	-534/-276, site 3	GCTGGGCGAGTTAGAACTGA	GCCCGGGATTAAAACTACACTG
*PAX2*	4842, NM_000278	-115/8, site 4	TGGCGAATCACAGAGTGGTGGAAT	GCTCCCGGTGTGTGTCTCTCTAAAA
*PAX2*	4842, NM_000278	-28/141, site 5	GGGCTTTGCAGCTTTTAGAGAG	GGAAAAGGCAGGCGCACG
*GNB2L1*	33871, NM_006098	-259/-134, GNB2L1	ACTTCACCTCTTTCGCTTCTCGCT	ACAGTCCCGTCTTCCGTACAACAA

### SMAD2 or SMAD3 protein-protein co-immunoprecipitation

HEK293 cells were transiently transfected with the PAX2 expression construct, *pCMV-PAX2b*, using FuGENE. After 42h of transfection, the cells were treated with TGF-β1 for 6h to activate SMAD2 and SMAD3. The cells were then lysed in non-denaturing lysis buffer supplemented with protease inhibitors (Sigma-Aldrich). The protein lysates (500 μg per antibody) were precleared for 1h at 4°C using Dynabeads® Protein G (Invitrogen), followed by immunoprecipitation using mouse anti-SMAD2/3 (Santa Cruz Biotechnology) or control IgG antibodies (Sigma-Aldrich), which was carried out overnight at 4°C. This was followed by incubation with 50 μl Dynabeads for 4h at 4°C, and then the magnetically captured protein complexes on Dynabeads were washed twice with lysis buffer, and the protein complexes eluted by boiling in Laemmli sample buffer (0.24 M Tris-HCl pH 6.8, 10% SDS, 40% Glycerol, 20% β-mercaptoethanol, 0.02% Bromophenol blue) under reducing conditions. Proteins were then resolved by SDS-PAGE and analyzed by Western blot to investigate SMAD2- or SMAD3-PAX2 or PAX6 interactions. Following the detection of PAX2, the Western blot membrane was stripped and incubated with an anti-SMAD2/3 antibody (1:1000, Santa Cruz Biotechnology) to detect SMAD2/3 proteins, and then the membrane was directly re-probed with anti-PAX6 antibody (1:100, Developmental Studies Hybridoma Bank) to detect PAX6 protein.

### *PAX2* autoregulation assays

In co-transfection assays, HEK293 cells were transiently transfected with one of two *PAX2* expression constructs (either CMV-PAX2b (PAX2b), or CMV-PAX2c (PAX2c) [67], together with the *PAX2* promoter-reporter plasmid, pGPxp2. Cells were co-transfected with a total of 600 ng of plasmids, comprising 150 ng of either pCMV-PAX2b or pCMV-PAX2c, and 150 ng of pGPxp2 with the remainder of the transfected plasmid being made up to an amount of 575 ng using pCMV empty vector (pCMV-EV). In some transfections up to 425 ng of either PAX2 expression vector, or PAX2 promoter-reporter construct was transfected, while the concentrations of either the expression, or the reporter construct, respectively, were maintained at 150 ng, and the amount of pCMV-EV was adjusted accordingly to make the total plasmid up to 575 ng. In addition, pCMV- β-gal (25 ng) was also included in all transient transfections to normalize the data to the β-gal value. Control transfections included cells only, pGL2 basic vector alone, CMV-PAX2b or CMV-PAX2c alone, and PGL2-basic together with CMV-PAX2b or CMV-PAX2c, and these were included in each experiment. The luciferase activity from “cells only”, and “pGL2 basic vector alone” was undetectable and is not shown. Luciferase activity is represented in Relative Light Units (RLU) normalised to the β-gal value.

### Online software and statistical analysis

For qRT-PCR analysis, the qBase software (version 1.3.5, http://medgen.ugent.be/qbase/) was used. For identification of SMAD binding sites in the *PAX2* promoter, the online web-based tools ConTra v.2 and TRANSFAC were used. We used ConTra v.2 for visualization of SMAD1 or SMAD3 binding sites in a region 3.5Kb upstream, or the 5’UTR region of the human *PAX2* gene on the sequence, NM_003987, with a core of 0.90, and similarity matrix of 0.75. Subsequently, we used ConTra v.3 for visualization of SMAD3 binding sites in the same 3.5Kb region of the same sequence (NM_003987), and a core of 0.95 and similarity matrix of 0.85, and this identified only the SMAD3 binding site previously identified at position -220 upstream of the TSS, which was conserved in species from humans to zebrafish.

## SUPPLEMENTARY MATERIALS FIGURES


