# Supplementary material for: SMAD proteins directly suppress PAX2 transcription downstream of transforming growth factor-beta 1 (TGF-β1) signalling in renal cell carcinoma
Source: Oncotarget. 2018 Jun 1;9(42):26852–67. doi: 10.18632/oncotarget.25516 (PMC6003550; doi:10.18632/oncotarget.25516)
Supplement: Supplementary file 1 [file oncotarget-09-26852-s001.pdf]

SMAD proteins directly suppress *PAX2* transcription downstream of transforming growth factor-beta 1 (TGF-β1) signalling in renal cell carcinoma

SUPPLEMENTARY MATERIALS

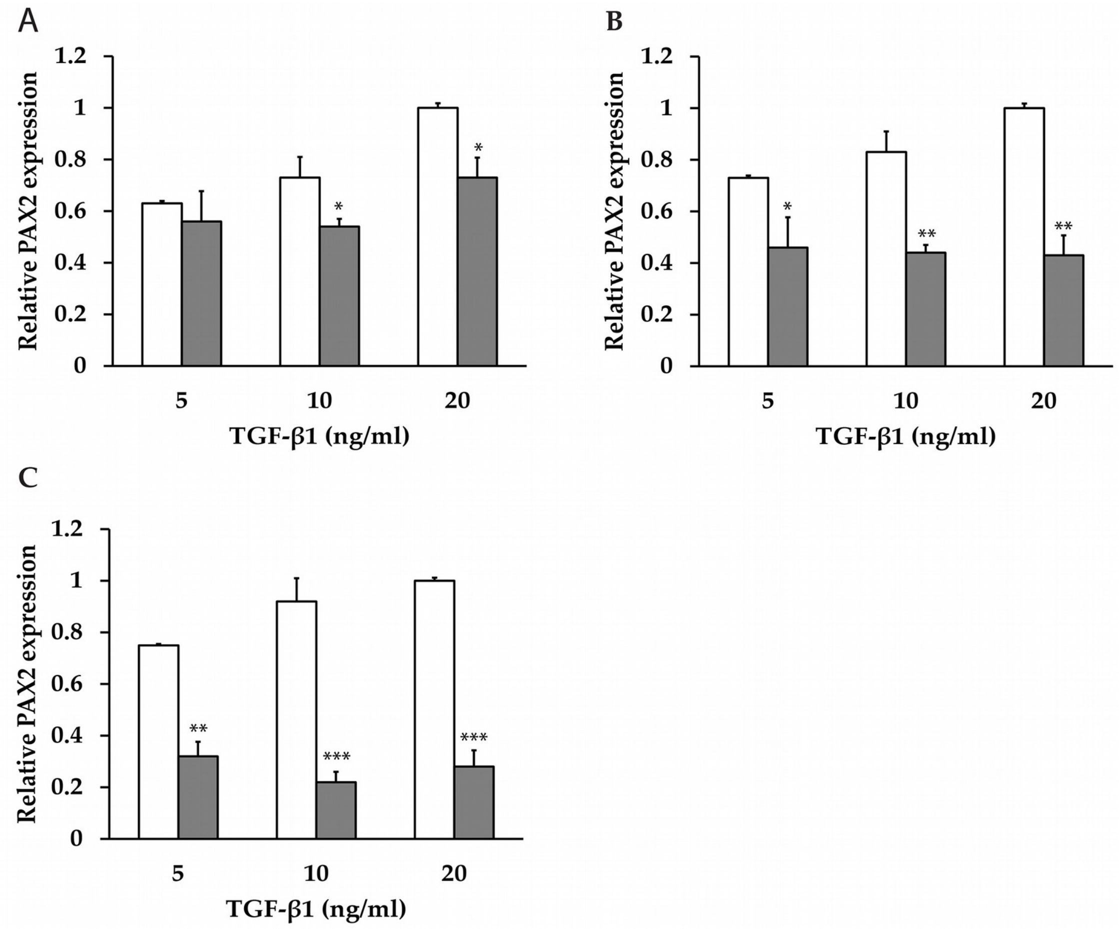

Supplementary Figure 1: Analysis of *PAX2* mRNA expression levels following treatment of 786-O cells with different concentrations of TGF-β1 for different time intervals (6, 12, 24h). The graphs show quantitative real-time PCR analysis of relative *PAX2* mRNA levels following treatment of 786-O cells with 5 ng/ml, 10 ng/ml and 20 ng/ml TGF-β1 for **(A)** 6 h **(B)** 12 h and **(C)** 24 h. The expression profile of *PAX2* was normalized to the three most stable housekeeping genes. The data were analyzed by Student's *t* test using GraphPad Prism 5.01 and represented as the mean ± S.E.M of three experiments. *, $p < 0.05$, **, $p < 0.01$, ***, $p < 0.001$ versus vehicle treated cells. White bars represent vehicle treated control cells and grey bars represent TGF-β1 treated cells.

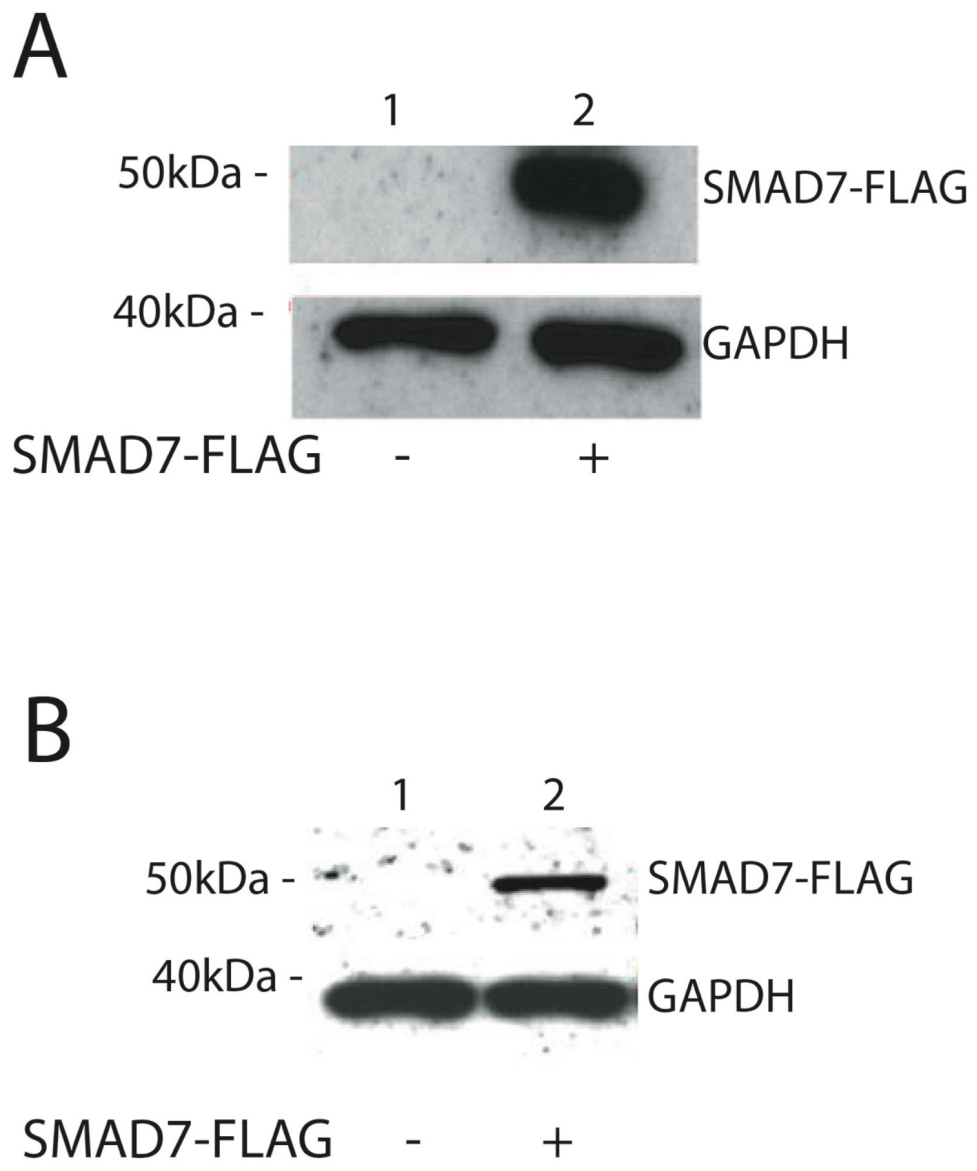

Supplementary Figure 2: Western blot analysis of confirmation of SMAD7 expression following transient transfection of **(A)** 786-O cells, and **(B)** HEK-293 cells using a SMAD7-FLAG expression construct and detection using anti-FLAG antibody. GAPDH served as a loading control.

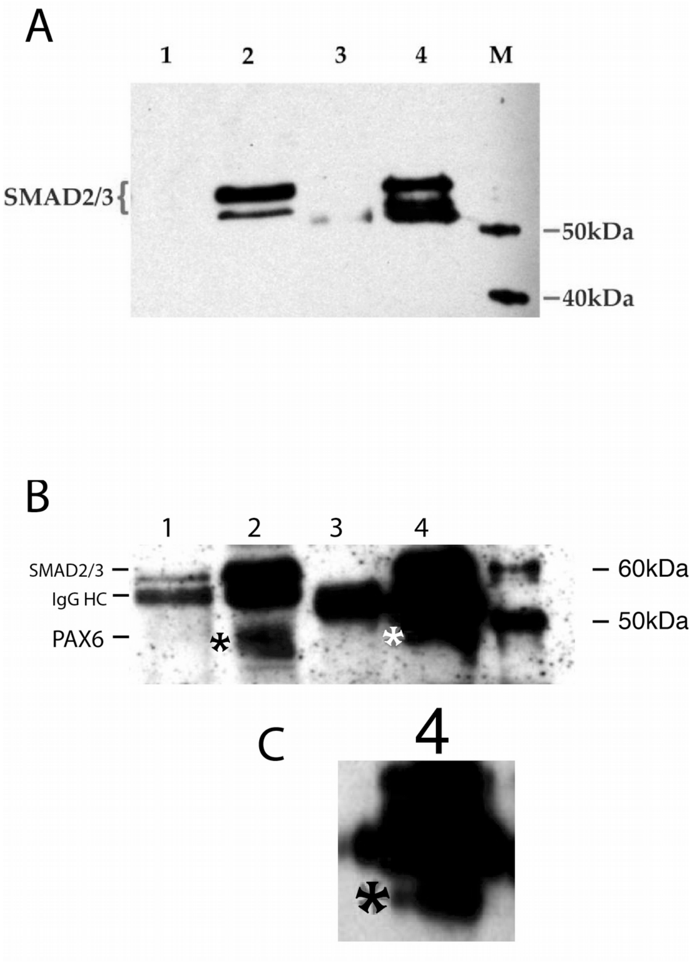

Supplementary Figure 3: Investigation of protein-protein interactions between SMAD2/3 and PAX6. **(A)** First, to confirm that the anti-SMAD2/3 antibody had immunoprecipitated SMAD2/3 protein as a positive control for immunoprecipitation, the same Western blot membrane that was used in Figure 4B was stripped and re-probed using an anti-SMAD2/3 antibody. Lane 1: Eluate from beads used for pre-clearing the lysate. Lane 2: Input. Lane 3: Eluate from beads, which captured the protein complexes incubated with anti-IgG antibody. Lane 4: Eluate from beads, which captured the protein complexes incubated with anti-SMAD2/3 antibody. These results show that the anti-SMAD2/3 antibody was competent in immunoprecipitation, because SMAD2/3 proteins were detected in lanes 2 and 4. **(B)** The same blot used for the SMAD protein detection was then directly re-probed (without stripping) using an anti-PAX6 antibody. The lanes were as before, and the results suggested that the anti-SMAD2/3 antibody had co-immunoprecipitated the known interactor, PAX6 (*), as seen in lane 4 by comparison to input in lane 2. Note that the signal from the previous SMAD2/3 Western blot carried over and was still detected in lanes 2 and 4 (indicated as “SMAD2/3”). the IgG heavy chain (IgG HC) signal was also detected in lanes 1, 3 and 4. **(C)** A magnified image of a shorter exposure of lane 4 is shown at the bottom of the figure for comparison to B).
